# Surveillance for Invasive Meningococcal Disease in Children, US–Mexico Border, 2005–2008^1^

**DOI:** 10.3201/eid1703.101254

**Published:** 2011-03

**Authors:** Enrique Chacon-Cruz, David E. Sugerman, Michele M. Ginsberg, Jackie Hopkins, Jose Antonio Hurtado-Montalvo, Jose Luis Lopez-Viera, Cesar Arturo Lara-Muñoz, Rosa M. Rivas-Landeros, Maria Luisa Volker, John A. Leake

**Affiliations:** Author affiliations:, General Hospital of Tijuana, Tijauna, Mexico (E. Chacon-Cruz, J.A. Hurtado-Montalvo, J.L. Lopez-Viera, C.A. Lara- Muñoz, R.M. Rivas-Landeros, M.L. Volker);; Centers for Disease Control and Prevention, Atlanta, Georgia, USA (D. Sugerman);; San Diego County Health and Human Services Agency, San Diego, California, USA (M.M. Ginsberg, J. Hopkins);; Rady Childrens Hospital, San Diego (J. Leake);; University of California, San Diego (J. Leake)

**Keywords:** Bacteria, Neisseria meningitidis, meningococcal disease, children, US–Mexico border, dispatch

## Abstract

We reviewed confirmed cases of pediatric invasive meningococcal disease in Tijuana, Mexico, and San Diego County, California, USA, during 2005–2008. The overall incidence and fatality rate observed in Tijuana were similar to those found in the US, and serogroup distribution suggests that most cases in Tijuana are vaccine preventable.

Invasive meningococcal disease (IMD) is caused by *Neisseria meningitidis.* Specific antibodies against the capsule are used to define the 13 known *N. meningitidis* serogroups (*1*). In the United States, *N. meningitidis* is a leading cause of bacterial meningitis (*2**,**3*). According to the provisional Active Bacterial Core Surveillance report of the Centers for Disease Control and Prevention, 1,050 cases of IMD were estimated to occur in 2008, with an overall incidence of 0.33/100,000 population and mortality rate of 0.03/100,000 population. Higher age-specific incidence and proportion of deaths occur in children and adolescents (*4*). In the United States, Active Bacterial Core Surveillance data show that serogroups B (0.11/100,000), C (0.11/100,000), and Y (0.08/100,000) are predominant (*5*).

IMD is a reportable condition in both the United States and Mexico. In 2006, the Mexican National Epidemiologic Surveillance System reported 60 cases in Mexico (population 105,790,700) for a nationwide rate of 0.056/100,000 (*6*). However, only a limited number of epidemiologic descriptions of IMD, primarily from outbreaks, are available from Mexico. For example, an outbreak of 753 cases was recorded during 1945–1949 in San Luis Potosi. Most cases were among infants and young children; serogroup data were not available (*7*).

Although physicians in Mexico at the US–Mexico border areas often encounter patients with symptoms highly compatible with IMD, diagnosis is not routinely culture-confirmed; this likely leads to underreporting. Serogroup-specific data on IMD are also lacking elsewhere throughout Mexico. The goals of our study were to compare hospital-based estimates of IMD in children and serogroup distribution at Tijuana General Hospital (TGH), Mexico, with a catchment population of nearly 200,000 children <17 years, to reported IMD cases in children in San Diego County (SDC), with a population of 723,600 children <17 years. (All demographic and serogroup data are listed in the Table.) This border is the most traversed international frontier in the world. We hypothesized that rates of IMD are underreported at TGH and that serogroup distribution is similar on both sides of the US–Mexico border.

**Table:** Characteristics of pediatric case-patients who had invasive meningococcal disease, Tijuana, Mexico, and San Diego County, California, USA, October 1, 2005–May 31, 2008*

Characteristics	No. (%) patients

Tijuana, n = 16	San Diego County, n = 13
Age, y		
<1	2 (12.5)	6 (46.2)
1–4	7 (43.8)	4 (30.8)
5–11	4 (25.0)	2 (15.4)
12–16	3 (18.8)	1 (7.7)
Male sex	8 (50.0)	9 (69.2)
Race/ethnicity		
Non-Hispanic white	0	6 (46.2)
Hispanic	16 (100)	3 (23.1)
Non-Hispanic black	0	2 (15.4)
Asian	0	0
Pacific Islander	0	0
Other/unknown	0	2 (15.4)
Month of onset		
November–February	12 (75.0)	7 (53.8)
March–June	2 (25.0)	4 (30.8)
July–October	2 (25.0)	2 (15.4)
Serogroup		
A	0	0
B	2 (12.5)	8 (61.5)
C	10 (62.5)	2 (15.4)
Y	2 (12.5)	1 (7.7)
W135	0	0
Not typeable	0	0
Not typed	2 (12.5)	2 (15.4)
Case-fatality rate	3 (18.8)	2 (15.4)

*Tijuana cases from hospital-based estimates and serogroup distribution at Tijuana General Hospital. San Diego County cases taken from those reported to the San Diego County Health and Human Services Agency through electronic laboratory notification or from infection control practitioners.

## The Study

During October 1, 2005–May 31, 2008, active surveillance for IMD was initiated at TGH among children <17 years of age. Blood or cerebrospinal fluid specimens, or both, were collected from all patients with suspected sepsis, meningitis, or purpura fulminans. Data on all pediatric IMD reported to the SDC Health and Human Services Agency through electronic laboratory notification or from infection control practitioners were retrospectively analyzed during the same period. Inclusion criteria as follows: per Centers for Disease Control and Prevention guidelines (*8*), we included only confirmed cases with sterile-site (blood/cerebrospinal fluid) isolation of *N. meningitidis*. At the SDC Public Health Laboratory, *N. meningitidis* isolates were identified by using API NH (bioMérieux, La Bolme-les-Grottes, France) and serogrouped by standard slide agglutination methods. At TGH, isolates were serogrouped by standard latex well agglutination methods using Pastore*x* Meningitis kit (Alere Ltd, Stockport, UK). Six case-patients from TGH and 7 from SDC with purpura fulminans, disseminated intravascular coagulation, or both, lacked culture-proven *N. meningitidis* infection and were excluded from analysis. Because patients >17 years are not hospitalized in the Department of Pediatrics at TGH, they were also excluded from the SDC data.

Clinical, microbiologic, and demographic data from TGH and SDC were analyzed by using STATA, Version 9.2 (StataCorp, LP, College Station, TX, USA). Pearson χ^2^ and Fisher exact tests were performed; p values <0.05 were defined as significant.

**Figure 1:**
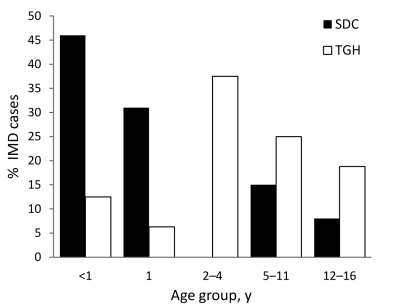
Cases of invasive meningococcal disease, by case-patient age group, Tijuana General Hospital (TGH), Tijuana, Mexico, and San Diego County (SDC), California, USA, October 1, 2005–May 31, 2008.

During the study period, a total of 29 pediatric cases of IMD were diagnosed, 16 at TGH (an estimated 3.08 annual cases/100,000 children <17 years) and 13 in SDC (0.69 annual cases/100,000 children <17 years). Children <5 years accounted for most IMD cases at both sites: 9 cases at TGH, and 10 in SDC (p = 0.24) (Figure 1). Of the 29 case-patients, 11 children were 1–4 years of age, and 8 were infants <1 year of age. Children <1 year of age accounted for 2 cases at TGH and 6 cases in SDC (p<0.05), with most infections in SDC caused by serogroup B. A slight male predominance (55.2%) was observed on both sides of the border, and 65.5% were diagnosed during November–February.

**Figure 2:**
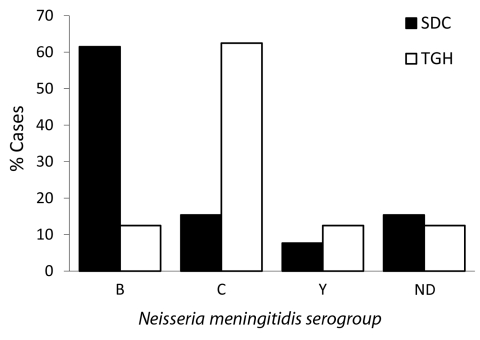
Cases of invasive meningococcal disease, by serogroup, Tijuana General Hospital (TGH), Tijuana, Mexico, and San Diego County (SDC), California, USA, October 1, 2005–May 31, 2008. ND, typing not done.

Overall, serogroup C was most commonly identified among the 29 cases (41.4%), followed by B (34.5%) and Y (10.3%); another 13.8% of cases were not serogrouped (2 cases each at TGH and SDC). A significant difference in serogroup was observed by site: serogroup C was most commonly identified at TGH (62.5%), whereas serogroup B was most common in SDC (61.5%) (Figure 2) (p = 0.005).

Of the 29 IMD case-patients from TGH and SDC, 5 children died, including 3 from TGH and 2 in SDC. Four of those who died were <5 years of age, and 1 child was 15 years of age. One fatal infection was known to be potentially vaccine preventable (caused by serogroup C), but the organisms in the other 4 deaths were not serogrouped.

## Conclusions

Documented reports of confirmed IMD in Mexico are rare (*6*), resulting in an assumption that incidence is extremely low. However, other infectious diseases, including tuberculosis, HIV/AIDS, and hepatitis A, B, and C, are common in this border region and often occur at higher rates than elsewhere in the United States (*9*) This surveillance project describes active hospital-based surveillance and serogroup distribution of IMD in children on both sides of the US–Mexico border. The age and serogroup distribution differed greatly between sites, with SDC demonstrating more infant cases and serogroup B, while TGH demonstrated more child and adolescent cases and serogroups C and Y.

This study suggests that rates of IMD at TGH, and presumably Tijuana and elsewhere in Mexico, may be substantially higher than reported. During the study period, vaccine-preventable serogroups were more common in TGH than in SDC. This finding has potential implications for immunization with the meningococcal vaccines containing serogroups C and Y in Mexico. In the United States, the quadrivalent conjugated meningococcal vaccine is recommended for all persons 11–18 years of age and is indicated for persons 2–55 years of age who are at increased risk for IMD (*10**,**11*). This vaccine might have benefits in Tijuana in terms of carriage of the bacteria and reduction in serogroup-specific IMD incidence, effects which have been demonstrated elsewhere (*12**,**13*). Widespread meningococcal vaccination has not yet been introduced in Tijuana or elsewhere in Mexico, although the monovalent m**eningococcal** C conjugate vaccine has been licensed in Mexico. This study suggests that a substantial number of IMD cases might have been prevented with quadrivalent conjugated meningococcal vaccine (75%) or monovalent serogroup C vaccine (63%). A recent study has shown that monovalent serogroup C vaccination administered to children <2 years of age could be effective in preventing IMD among infants (*14*).

This investigation was limited in several aspects, however. Tijuana serogroup data was only available for TGH (the city’s indigent tertiary care referral center), which likely led to an underestimation of the number of pediatric IMD cases. Even though data were reviewed for nearly 3 years, the relatively small geographic area resulted in a small sample size, which limits generalizations.

IMD is likely to occur at a higher rate than previously reported in Tijuana. The overall incidence and fatality rate observed for TGH cases were similar to rates in the United States, and serogroup distribution at TGH indicates that most IMD cases in Tijuana are vaccine preventable. Establishment of a binational IMD surveillance system could provide substantial benefit in improving IMD control potentially leading to vaccination strategies in Mexico’s northern border region, and perhaps elsewhere. Further IMD surveillance studies including binational systems are needed to better define the epidemiology of IMD in the northern border and other regions of Mexico and determine appropriate vaccination policies.
